# Value of thromboelastography in managing hypercoagulopathy in intensive care

**DOI:** 10.1186/cc14420

**Published:** 2015-03-16

**Authors:** J Aron, A Gibbon, C Ward, J Ball

**Affiliations:** 1 St George's Hospital, London, UK

## Introduction

This aim of this analysis is to explore the use of thromboelastography (TEG) in the management of hypercoagulation in the ICU. TEG allows the assessment of whole blood coagulation and fibrinolysis and hence can identify patients who are hypercoagulable.

## Methods

A prospective audit of TEG tests performed on patients being treated on a general surgical and medical ICU was conducted over a 2-month period.

## Results

**Figure 1:**
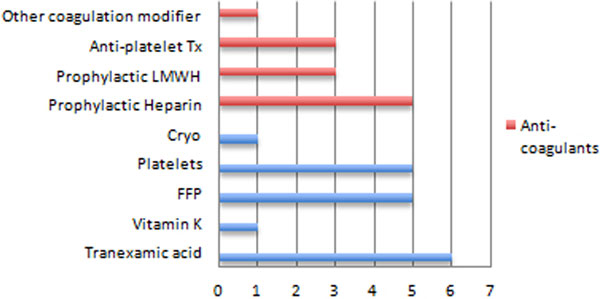
**Coagulation modification prior to TEG analysis in hypercoagulable patients**.

Twenty-one out of 78 patients (26.9%) had one or more TEG criteria consistent with hypercoagulopathy. Admission diagnoses included trauma (37%), haemorrhage (23%), postoperative (23%) and sepsis (14.3%). Sixty-two per cent of patients with a primary diagnosis of trauma were in a hypercoaguable state. Hypercoagulopathy was suggested by an abnormally short R time in 16 patients (76%), an abnormal alpha angle in 17 cases (81%), a maximum amplitude >74 mm in nine cases (43%) and a high LY30 in one case. Procoagulant treatment was given to seven patients and five patients had received no coagulation modification prior to testing (Figure 1). Eight patients were receiving prophylactic anticoagulation and only one was receiving treatment-dose anticoagulation. A change in management as a result of performing TEG was documented in 14 of these 21 patients. No further blood products were administered in all cases and anticoagulation was commenced or increased in four cases.

## Conclusion

Hypercoagulopathy was present in 27% of patients. One-third of these patients had recently received prothrombotic therapy indicating a possible iatrogenic aetiology. TEG analysis resulted in cessation of prothrombotic drug and blood product administration in all cases. Further research is required to determine whether titrated anticoagulation treatment to normalise the TEG profile in these patients would be beneficial.
